# Supplementary material for: Investigation of the Stability and Hydrogen Evolution Activity of Dual-Atom Catalysts on Nitrogen-Doped Graphene
Source: Nanomaterials (Basel). 2022 Jul 25;12(15):2557. doi: 10.3390/nano12152557 (PMC9332772; doi:10.3390/nano12152557)
Supplement: Supplementary file 1 [file nanomaterials-12-02557-s001.zip › nanomaterials-1828288-supplementary.pdf]

Supplementary Material

Investigation of the Stability and Hydrogen Evolution Activity of Dual-Atom Catalysts on Nitrogen-Doped Graphene

Qiansong Zhou [1,2], Meng Zhang [3], Beien Zhu [1,4,*] and Yi Gao [1,4,*]

[1] Key Laboratory of Interfacial Physics and Technology, Shanghai Institute of Applied Physics, Chinese Academy of Sciences, Shanghai 201800, China; zhouqiansong@sinap.ac.cn

[2] University of Chinese Academy of Sciences, Beijing 100049, China

[3] Department of Physics, School of Physical Science and Technology, Ningbo University, Ningbo 315211, China; zhangmeng733@126.com

[4] Interdisciplinary Research Center, Zhangjiang Laboratory, Shanghai Advanced Research Institute, Chinese Academy of Sciences, Shanghai 201210, China

* Correspondence: zhubeien@zjlab.org.cn (B.Z.); gaoyi@zjlab.org.cn (Y.G.)

Methodologies

We calculated the formation energy of two types of vacancy sites in graphene substrate structures to determine their stability (E_f) [54]:

$$E_f = E_{sub} + x\mu_C - E_G - y\mu_N \quad (1)$$

In the formula, E_{sub} is the total energy of $SVGN_3$ or 4N−doped double-vacancies graphene ($DVGN_4$); E_G is the total energy of pristine graphene substrate structure; x is the number of carbon atoms that were removed from the pristine graphene; μ_C is the chemical potential of carbon, defined as the total energy of each carbon atom in the pristine graphene; y is the number of embedded N atoms; μ_N is the chemical potential of nitrogen (defined as half of the total energy of the N_2 molecule). As the formation energy of $SVGN_3$ is 3.50 eV [55] and that of $DVGN_4$ is 3.88 eV, we will choose $SVGN_3$ as the study object.

Tables

Table S1. Testing the binding energy (E_b) under several functional (PBE, RPBE, PBE+D3) for 3d elements.

	E_b/eV(PBE)	E_b/eV(RPBE)	E_b/eV(PBE+D3)
TiG	-1.98	-1.28	-1.98
$TiGN_3$	-6.29	-5.94	-6.45
Ti_2GN_3	-9.92	-8.90	-9.92
VG	-1.20	-0.84	-1.20
VGN_3	-5.80	-5.32	-5.80
V_2GN_3	-7.79	-6.84	-7.79
CrG	-0.31	-0.09	-0.31
$CrGN_3$	-3.73	-3.25	-3.73
Cr_2GN_3	-5.70	-4.71	-5.70
MnG	-0.23	0.04	-0.27
$MnGN_3$	-3.98	-3.51	-3.98
Mn_2GN_3	-5.75	-3.99	-5.75
FeG	-1.17	-0.49	1.13
$FeGN_3$	-4.57	-3.67	-4.82
Fe_2GN_3	-7.21	-4.95	-7.15
CoG	-1.24	-0.99	-1.17
$CoGN_3$	-4.96	-4.65	-4.89
Co_2GN_3	-7.07	-6.81	--6.93
NiG	-1.52	-1.01	-1.52
$NiGN_3$	-4.48	-3.90	-4.26
Ni_2GN_3	-6.58	-5.76	-6.56
CuG	-0.29	0.00	-0.29
$CuGN_3$	-3.12	-2.59	-3.12
Cu_2GN_3	-4.54	-3.83	-4.50

Table S2. The formation energy of $SVGN_3$ and $DVGN_4$

	$SVGN_3$	$DVGN_4$
formation energy(eV)	3.50[55]	3.88

Table S3. The zero-point energy (ZPE), and entropic correction (TS, T = 298.15K) of H_2.

Species	ZPE/eV	TS/eV
H_2	0.269	0.404

Table S4. The adsorption energy (ΔE_{H^*}), zero-point energy difference (ΔE_{ZPE}), and entropic correction difference (TΔS_{H^*}, T = 298.15K), Gibbs free energy (ΔG_{H^*}) of H−SACs at a potential U = 0. The ZPE and entropy of the substrate are negligible.

	ΔE_{H^*}/eV	ΔE_{ZPE}/eV	TΔS_{H^*}/eV	ΔG_{H^*}/eV
Ti	-0.995	0.0885	-0.189	-0.72
V	-0.665	0.0245	-0.182	-0.46
Cr	-0.595	0.0125	-0.172	-0.41
Mn	-0.485	0.0035	-0.196	-0.29
Fe	-0.675	0.0175	-0.172	-0.49
Co	-0.175	0.0415	-0.180	0.05
Ni	-0.185	0.0495	-0.191	0.06
Cu	0.095	0.0425	-0.032	0.17
Zr	-0.965	0.0025	-0.171	-0.79
Nb	-1.055	0.0205	-0.181	-0.85
Mo	-0.975	0.0385	-0.187	-0.75
Tc	-0.765	0.0425	-0.187	-0.54
Ru	-0.495	0.0545	-0.191	-0.25
Rh	-0.675	0.0765	-0.132	-0.47
Pd	-0.355	0.0755	-0.194	-0.09
Ag	0.365	0.0235	-0.175	0.56
Hf	-1.425	0.0095	-0.174	-1.24
Ta	-1.375	0.0265	-0.181	-1.17
W	-1.425	0.0435	-0.186	-1.20
Re	-1.335	0.0505	-0.187	-1.10
Os	-0.975	0.0405	-0.181	-0.75
Ir	-1.425	0.0805	-0.194	-1.15
Pt	-1.335	0.0775	-0.192	-1.07
Au	-0.935	0.0645	-0.186	-0.68

For H−Co_1−$SVGN_3$ (T=298.15K), (1) Geometry optimization is performed first, followed by vibration frequency calculation (releasing H atom; fixing Co, C, and N atoms), and finally, VASPKIT post-processing software calculates the zero-point energy (ZEP = 0.176eV) and entropy (TS = 0.022eV) of the adsorbed hydrogen. (2) Similarly, the zero-point energy (ZEP = 0.1345 eV) and entropy (TS = 0.202 eV) of hydrogen in the gas phase H2 molecule were calculated. (3) We take the difference between the zero-point energy and the entropy of the adsorbed hydrogen and gas phase hydrogen to get $\boldsymbol{\Delta E_{ZPE}}$ = 0.0415eV and $\boldsymbol{T\Delta S_{H^*}}$ = -0.180eV. (4) According to the formula $\Delta \boldsymbol{G_{H^*}} = \Delta \boldsymbol{E_{H^*}} + \Delta \boldsymbol{E_{ZPE}} - \boldsymbol{T\Delta S_{H^*}}$, the Gibbs free energy of H adsorption in the H−Co_1−$SVGN_3$ system is 0.05eV, which is consistent with previous work.[38]

Table S5. The adsorption energy (ΔE_{H^*}), zero-point energy difference (ΔE_{ZPE}), and entropic correction difference ($T\Delta S_{H^*}$, T = 298.15K), Gibbs free energy (ΔG_{H^*}) of H−DACs at a potential U = 0. The ZPE and entropy of the substrate are negligible.

	ΔE_{H^*}/eV	ΔE_{ZPE}/eV	$T\Delta S_{H^*}$/eV	ΔG_{H^*}/eV
Ti	-0.595	0.1595	-0.202	-0.23
V	-1.335	0.0415	-0.185	-1.11
Cr	-0.605	0.0165	-0.178	-0.41
Mn	-0.345	0.0325	-0.157	-0.16
Fe	-0.545	0.0505	-0.191	-0.30
Co	-0.695	0.0435	-0.184	-0.47
Ni	-0.935	0.0425	-0.182	-0.71
Cu	-1.105	0.0655	-0.188	-0.85
Zr	-0.965	0.0035	-0.169	-0.79
Nb	-1.505	0.0365	-0.189	-1.28
Mo	-0.655	0.0165	-0.179	-0.46
Tc	-1.245	0.0545	-0.192	-1.00
Ru	-0.865	0.0345	-0.167	-0.66
Rh	-0.605	0.0365	-0.191	-0.38
Pd	-0.685	0.0405	-0.189	-0.46
Ag	-0.665	0.0455	-0.186	-0.44
Hf	-1.215	0.0065	-0.169	-1.04
Ta	-1.395	0.0385	-0.189	-1.17
W	-1.255	0.0275	-0.185	-1.04
Re	-0.335	0.0575	-0.189	-0.09
Os	-0.595	0.0605	-0.177	-0.36
Ir	-0.805	0.0615	-0.167	-0.58
Pt	-0.695	0.0545	-0.170	-0.47
Au	-0.665	0.0695	-0.188	-0.40

Figures

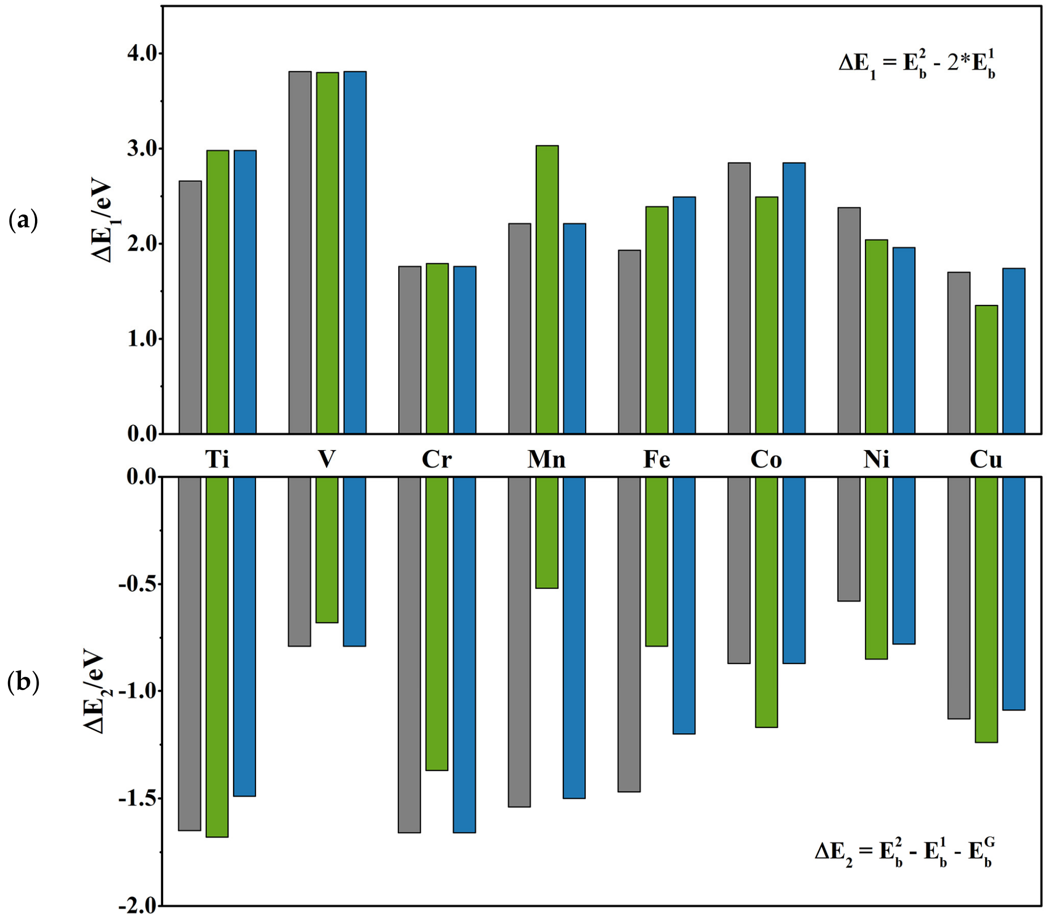

Figure S1. (a) ΔE_1 is the difference between E_b^2 and $2 * E_b^1$. (b) ΔE_2 is the difference between E_b^2 and $(E_b^1 + E_b^G)$. (Gray, green, and blue histograms represent for PBE, RPBE, and PBE+D3, respectively.)

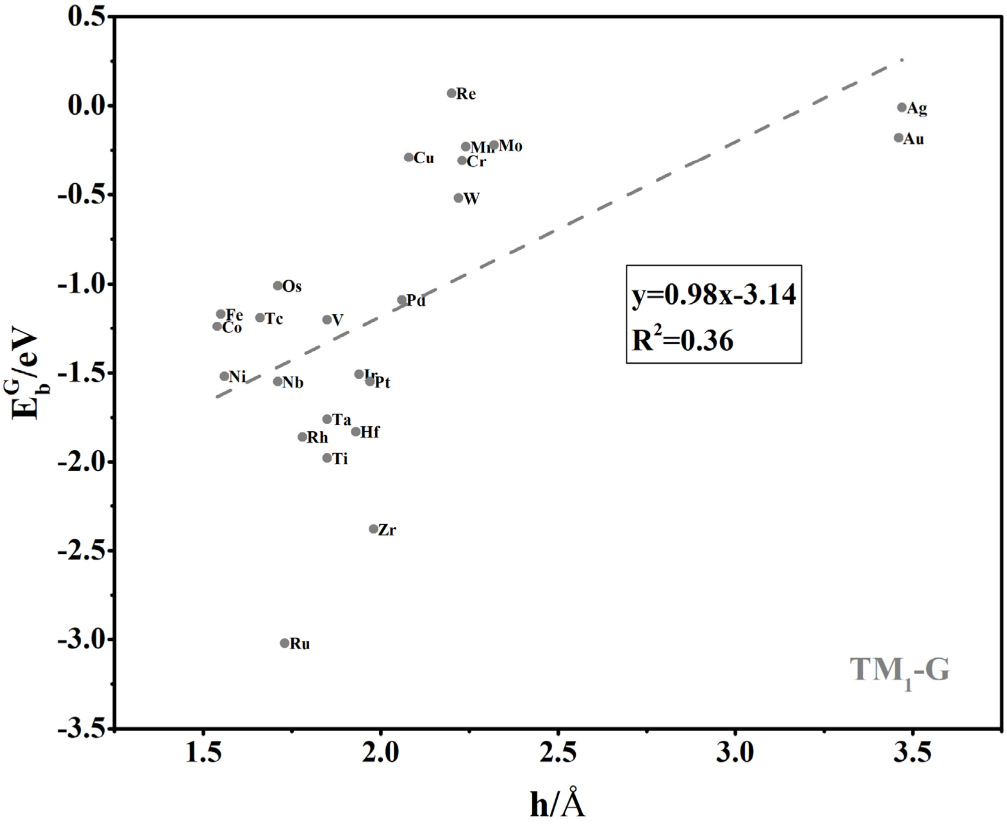

Figure S2. Correlation between the height (h) from the M1 to the pure graphene and binding energy (E_b^G) in TM_1−G.

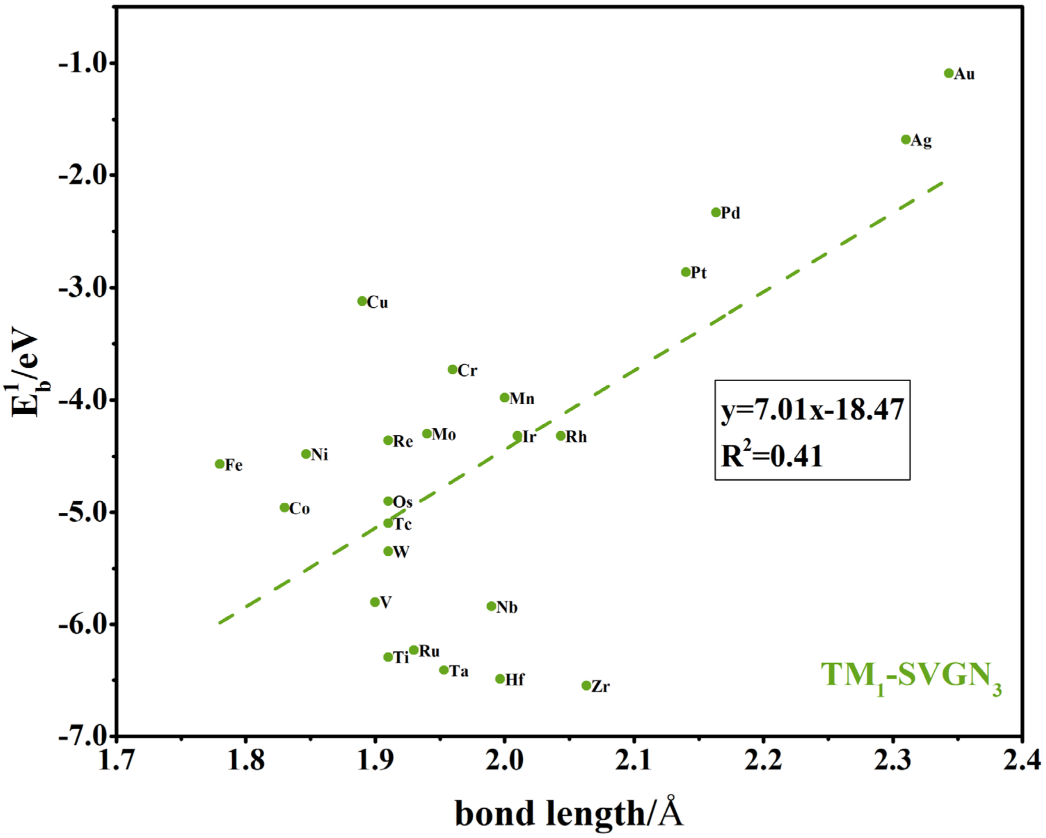

Figure S3. Correlation between the average distance (d_{M1-Nx}) from the anchored M1 to the N atom and binding energy (E_b^1) in TM_1-SVGN_3.

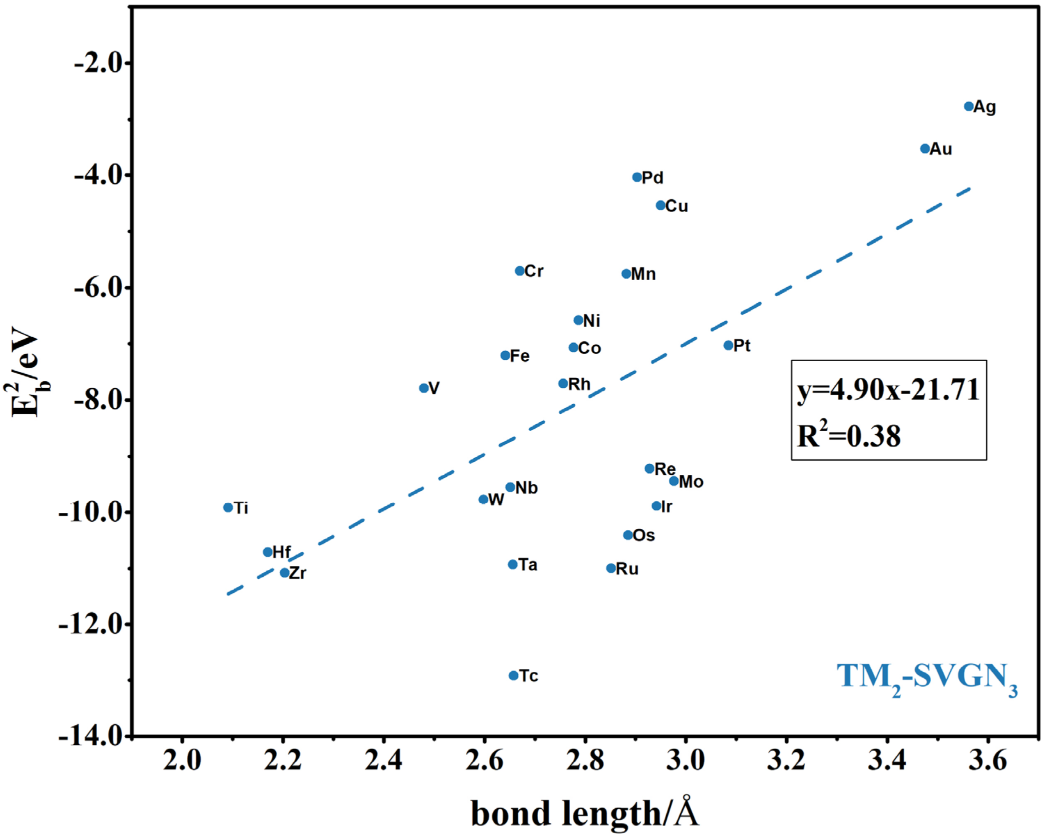

Figure S4. Correlation between the average distance (d_{M1-Nx} and d_{M2-Nx}) from the dual-atoms (M1 and M2) to the N atom and binding energy (E_b^2) in TM_2−$SVGN_3$.
